# Supplementary material for: Complex‐centric proteome profiling by SEC‐SWATH‐MS
Source: Mol Syst Biol. 2019 Jan 14;15(1):e8438. doi: 10.15252/msb.20188438 (PMC6346213; doi:10.15252/msb.20188438)
Supplement: Supplementary file 7 — Dataset EV6 [file MSB-15-e8438-s007.zip › feature_plots_bioplex/Q8NFB2.pdf]

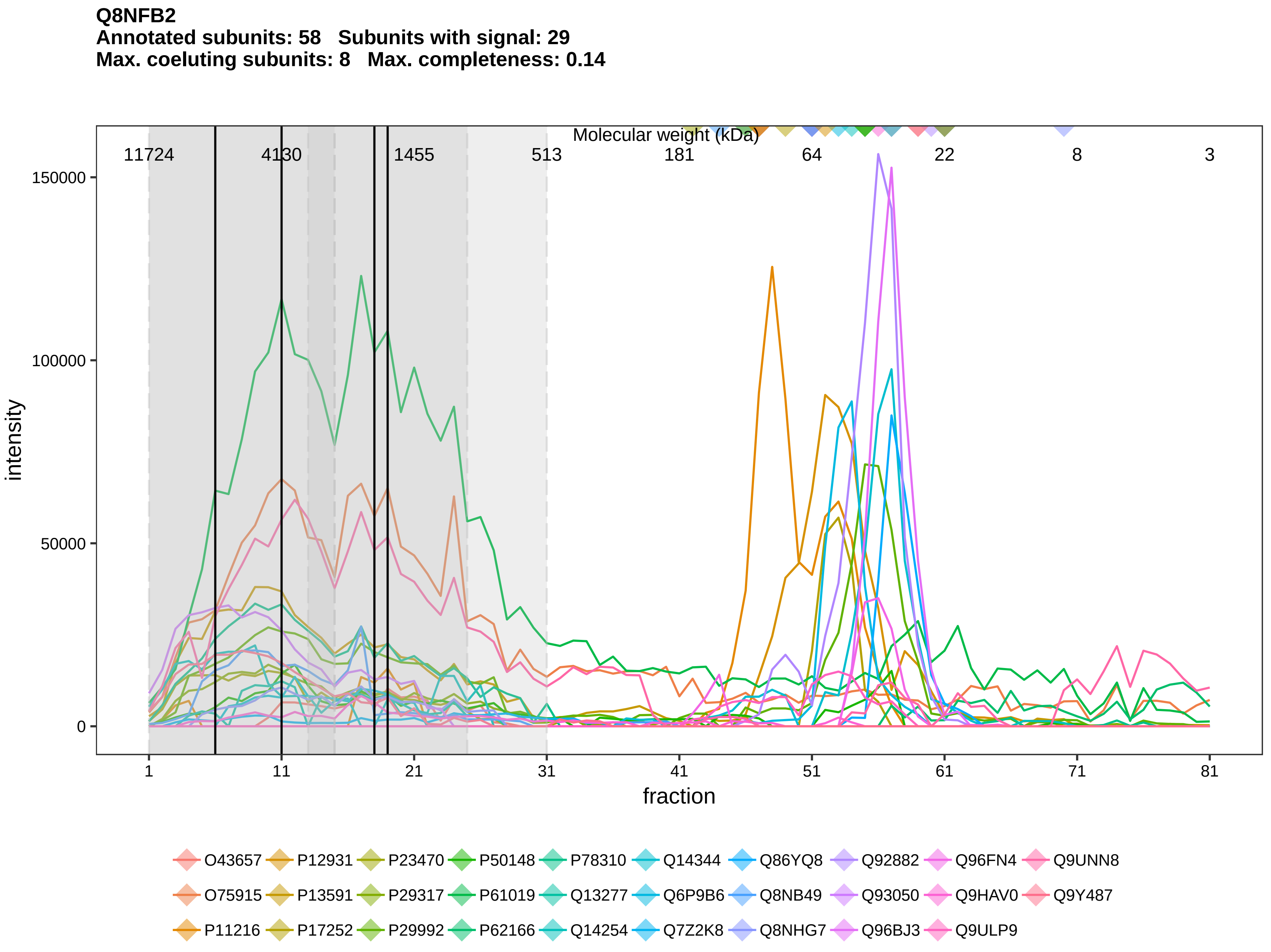
Q8NFB2
Annotated subunits: 58 Subunits with signal: 29
Max. coeluting subunits: 8 Max. completeness: 0.14
Molecular weight (kDa)
11724
4130
1455
513
181
64
22
8
3
intensity
150000
100000
50000
0
fraction
1
11
21
31
41
51
61
71
81
O43657
P12931
P23470
P50148
P78310
Q14344
Q86YQ8
Q92882
Q96FN4
Q9UNN8
O75915
P13591
P29317
P61019
Q13277
Q6P9B6
Q8NB49
Q93050
Q9HAV0
Q9Y487
P11216
P17252
P29992
P62166
Q14254
Q7Z2K8
Q8NHG7
Q96BJ3
Q9ULP9
